# Systemic N‐Acylethanolamines and Other Lipid Mediators Are Associated With NSAID‐Exacerbated Respiratory Disease

**DOI:** 10.1111/cea.70145

**Published:** 2025-09-18

**Authors:** Viljami E. Salmi, Annina Lyly, Mikko Neuvonen, Elmo Saarentaus, Mikko Niemi, Sanna Toppila‐Salmi

**Affiliations:** ^1^ Department of Allergology, Inflammation Center Helsinki University Hospital and University of Helsinki Helsinki Finland; ^2^ Department of Otorhinolaryngology University of Eastern Finland, Joensuu and Kuopio Finland; ^3^ Department of Otorhinolaryngology‐Head and Neck Surgery University of Helsinki and Helsinki University Hospital Helsinki Finland; ^4^ Individualized Drug Therapy Research Program University of Helsinki Helsinki Finland; ^5^ Institute for Molecular Medicine Finland (FIMM), University of Helsinki Helsinki Finland; ^6^ Department of Clinical Pharmacology University of Helsinki Helsinki Finland; ^7^ HUS Diagnostic Center Helsinki University Hospital Helsinki Finland; ^8^ Department of Otorhinolaryngology, Wellbeing Services County of Pohjois‐Savo Kuopio Finland

**Keywords:** acetylsalicylic acid, AEA, arachidonic acid, asthma, lipid mediator, LTE4, N‐ERD, OEA

**Summary**

- A low dose of aspirin is sufficient to induce a change in N‐ERD patients' metabolism.
- Anti‐inflammatory and anti‐analgesic endocannabinoid‐related lipids AEA and OEA in plasma are significantly reduced in N‐ERD patients.

**Abbreviations**
11,12‐DHET: 11,12‐Dihydroxyeicosatrienoic acid
12‐HHT: 12‐hydroxyheptadecatrienoic acid
AA: arachidonic acid
AEA: arachidonoylethanolamide
ASA: acetylsalicylic acid
BLT2: leukotriene B4 receptor 2
CB2R: cannabinoid type 2 receptor
COX: cyclooxygenase
CRSwNP: chronic rhinosinusitis with nasal polyps
CYP450: cytochrome P450
EPA: eicosapentaenoic acid
FADS2: fatty acid desaturase 2
LOX: lipoxygenase
LT: leukotriene
NAE: N‐acylethanolamine
N‐ERD: NSAID‐exacerbated respiratory disease
OEA: oleoylethanolamide
PLA2: phospholipase A2
PPAR‐α: peroxisome proliferator‐activated receptor alpha

To the Editor,

Nonsteroidal anti‐inflammatory drug (NSAID)‐exacerbated respiratory disease (N‐ERD) is a common chronic airway inflammatory disease characterised by asthma, chronic rhinosinusitis with nasal polyps (CRSwNP) and hypersensitivity to NSAIDs, including acetylsalicylic acid (ASA, aspirin), posing a significant burden on respiratory health [1]. N‐ERD currently lacks reliable clinical laboratory tests, yet early diagnosis and management are essential to prevent disease progression [2]. The pathogenesis of N‐ERD has been linked to dysregulation of arachidonic acid (AA) metabolism, which affects levels of pro‐inflammatory lipid mediators such as leukotrienes and prostaglandins [1, 3]. ASA alters AA metabolism by inhibiting cyclooxygenase (COX) enzymes and redirecting lipid synthesis towards pro‐inflammatory leukotrienes [1]. Despite extensive research into eicosanoids in N‐ERD, the full spectrum of lipid mediators involved remains incompletely understood. In this study, we investigated the effect of a low dose of ASA (25 mg) on plasma and urine lipid mediator concentrations in N‐ERD patients compared with healthy controls, aiming to identify novel biomarkers and deepen understanding of disease mechanisms.

This study included eight consecutively recruited N‐ERD patients and seven age‐matched healthy controls (25–62 years). All subjects were Finnish females, selected to minimise demographic variability. N‐ERD was diagnosed based on typical symptoms after NSAID intake and confirmed with ASA challenge, lung function testing and clinical examination [1]. All patients had asthma and CRSwNP. Ethics approval and informed consent were obtained as part of the AirGOs‐Medical clinical trial (NCT03825757) [4].

Blood plasma and urine samples were collected after overnight fasting at baseline and 2 h after ingestion of 25 mg ASA (Primaspan, 50 mg tablet, Orion, Espoo, Finland). Although this initial dose did not provoke symptoms in all patients, it was part of a broader challenge that ultimately confirmed N‐ERD in each case (Figure 1A). Our aim was to detect metabolic changes while minimising the risk of severe respiratory reactions. Lipid mediators were quantified using liquid chromatography–tandem mass spectrometry, targeting 158 eicosanoids and related compounds, with 30 analytes quantified. Urine concentrations were normalised to creatinine levels.

**FIGURE 1:**
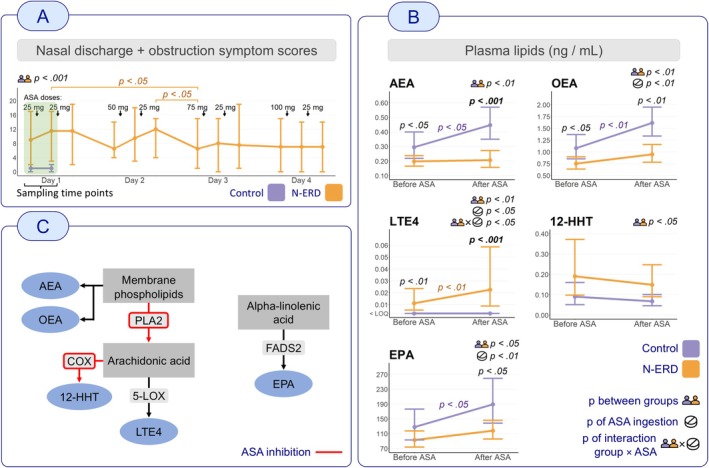
Panel A: Summed visual analogue scale (VAS) scores for nasal discharge and obstruction symptoms during ASA challenge. Plasma and urine samples were collected before and after the first dose (the green‐highlighted interval). Control symptom scores are available only for these time points. The figure shows the significance of differences between the N‐ERD and control groups at the first two time points, as well as within‐group changes in the N‐ERD group during the remainder of the desensitisation. Only *p* values < 0.05 are shown. Panel B: The line graphs illustrate the concentrations of lipid mediators in both groups (control and N‐ERD) before and 2 h after ingestion of ASA 25 mg. The graphs show the geometric means and 95% confidence intervals of the respective lipids. The *y*‐axis represents the concentrations (ng/mL) of lipid mediators in plasma. *p* values corresponding to specific comparisons (between‐group at a given time point and within‐group changes after ASA) are shown at the corresponding positions in the figures. *p* values for the fixed effects—representing the overall effects of group, ASA, and their interaction (i.e., different ASA effects between groups)—are displayed next to the names of the lipids. Only *p* values < 0.05 are shown and only lipids with a significant *p* value in the group comparison (*p* between groups) are shown. Additionally, Bonferroni correction was applied for 78 tests (26 lipids × 3 effects), resulting in a significance threshold of *p* < 0.00064. *p* values below this threshold are bolded. Panel C: Simplified metabolomic pathways of the lipids. The known inhibitory effects of ASA are shown. 12‐HHT, 12‐hydroxyheptadecatrienoic acid; 5‐LOX, 5‐lipoxygenase; AEA, arachidonoylethanolamide; ASA, acetylsalicylic acid; COX, cyclooxygenase; EPA, eicosapentaenoic acid; FADS2, fatty acid desaturase 2; LTE4, leukotriene E4; N‐ERD, NSAID‐exacerbated respiratory disease; OEA, oleoylethanolamide; PLA2, phospholipase A2.

Linear mixed‐effects models were used to evaluate the effects of group (N‐ERD vs. control), ASA intake (before vs. after) and their interaction (different ASA effects between groups) on individual lipid concentrations. Lipids with a significant *p* value (*p* < 0.05) in the group comparison were considered main findings and are presented in this letter.

Reduced plasma concentrations of the endocannabinoid‐related lipids arachidonoylethanolamide (AEA) and oleoylethanolamide (OEA) were associated with N‐ERD both at baseline and after ASA ingestion, compared with the control group (*p* < 0.01 for both) (Figure 1B). AEA and OEA are N‐acylethanolamines (NAEs), lipid mediators derived from membrane phospholipids, from which AA can also be released by phospholipase A2 (PLA2) (Figure 1C). AEA and OEA exert anti‐inflammatory effects via distinct pathways: AEA through cannabinoid type 2 receptors (CB2R), reducing pro‐inflammatory cytokines and OEA via peroxisome proliferator‐activated receptor alpha (PPAR‐α), which suppresses cytokine production, eosinophil recruitment and oxidative stress. Both NAEs are further metabolised by COX‐2, lipoxygenase (LOX) and cytochrome P450 (CYP450) enzymes into immunomodulatory metabolites [5]. Lower plasma AEA and OEA levels in N‐ERD suggest dysregulated NAE metabolism and impaired CB2R‐ and PPAR‐α‐mediated anti‐inflammatory signalling, with unchanged levels after ASA indicating impaired synthesis or downstream conversion. The endocannabinoid system is a conserved signalling network that modulates eicosanoid‐driven inflammation. CB2R activation reduces type 2 inflammation and AA levels, and its expression is upregulated in nasal polyp tissue of N‐ERD patients [6], suggesting a compensatory mechanism. Thus, reduced AEA and OEA levels may reflect negative feedback, explaining symptom severity, poor response to traditional management [7] and the potential role of biologics in N‐ERD [2].

Plasma leukotriene E4 (LTE4) levels were significantly higher in the N‐ERD group at both time points (*p* < 0.01). After ASA ingestion, LTE4 increased significantly in N‐ERD, while remaining below quantification in controls (group × ASA interaction *p* < 0.05) (Figure 1B). As a pro‐inflammatory mediator produced via the 5‐LOX pathway (Figure 1C), elevated LTE4 supports its role in N‐ERD pathophysiology and utility as a biomarker, as previously shown [3].

Plasma 12‐hydroxyheptadecatrienoic acid (12‐HHT) was significantly elevated in the N‐ERD group at both time points (*p* < 0.05) (Figure 1B). 12‐HHT, an AA‐derived mediator, suppresses allergic airway inflammation through leukotriene B4 receptor 2 (BLT2) activation (Figure 1C) [8]. Elevated levels may reflect ongoing chronic airway inflammation in N‐ERD and hold diagnostic potential.

Plasma eicosapentaenoic acid (EPA) was consistently significantly lower in the N‐ERD group (*p* < 0.05) (Figure 1B). EPA is an omega‐3 fatty acid produced from alpha‐linolenic acid by fatty acid desaturase 2 (FADS2) (Figure 1C), which is a precursor for pro‐resolving mediators, competes with AA for COX enzymes and suppresses inflammatory cytokines. Reduced levels may reflect dietary insufficiency or altered omega‐3 metabolism, potentially contributing to allergic airway inflammation in N‐ERD, as EPA has a protective role against the development of asthma and allergic diseases [9].

None of the measured lipid mediators in urine showed a statistically significant *p* value in the group comparisons. Detailed results also for other analysed lipids are available in the following repository: https://osf.io/eazn6/?view_only=6b8eff58edfc4c43a76d811680f93732.

To our knowledge, this is the first report demonstrating an association between reduced plasma concentrations of endocannabinoid‐related lipids AEA and OEA with N‐ERD. Impaired AEA or OEA signalling may contribute to heightened pain perception in N‐ERD patients [5], suggesting potential diagnostic and therapeutic implications. We also discovered other lipids that are potentially characteristic of N‐ERD metabolism. Further studies with a larger cohort are needed to confirm these findings.

## Author Contributions

S.T.‐S. and A.L.: recruitment of subjects, examination of subjects, sampling. M.Ni. and M.Ne.: laboratory experiments, data preparation. V.E.S.: data analysis, writing of manuscript, preparation of figures and tables. All authors have made substantial contributions to conception and design, or interpretation of data, and have been involved in revising the manuscript critically and have given final approval of the version to be published.

## Ethics Statement

Approval for the study was obtained from the National Drug Agency (Eudra CT 2017‐0015070‐42, KL/41/2018), Ethics Committee of Hospital District of Helsinki and Uusimaa (HUS/1801/2017).

## Consent

Written informed consent was obtained from all subjects.

## Conflicts of Interest

S.T.‐S. reports consultancies for ALK‐Abelló, AstraZeneca, Clario, ERT, GlaxoSmithKline, Novartis, Sanofi Pharma, Orion Pharma, Roche Products and grants from GlaxoSmithKline and Sanofi. All are outside the submitted work. A.L. reports a consultancy for AstraZeneca and a lecturing fee from the Finnish Lung Health Association. All are outside the submitted work. All other authors declare no conflicts of interest.

## Data Availability

Additional information about study methods and results is available in the following repository: https://osf.io/eazn6/?view_only=6b8eff58edfc4c43a76d811680f93732. The data that support the findings of this study are available upon request from the corresponding author.
